# Unexpected De Garengeot hernia in elective femoral hernia repair: A case report

**DOI:** 10.1016/j.ijscr.2025.111767

**Published:** 2025-08-08

**Authors:** Md. Shoieb Hossain Mridha, Ayman El-Shihaby, Lakshmanan Arunachalam

**Affiliations:** a Dept. of General Surgery, Doncaster and Bassetlaw Teaching Hospitals, NHS Trust, Doncaster, UK; b Dept. of Colorectal Surgery, Doncaster and Bassetlaw Teaching Hospitals, NHS Trust, Doncaster, UK

**Keywords:** Femoral hernia, De-Garengeot hernia, Groin swelling, CT scan, Surgical options

## Abstract

**Introduction:**

Femoral hernia is more common in women than in men, with the content of hernia sac typically being omentum or small bowel. De-Garengeot's hernia is a rare surgical condition where appendix is found inside a femoral hernia. Its unusual presentation often leads to an intraoperative diagnosis.

**Case presentation:**

We present the case of a 79-year-old woman who presented for an elective day case surgery for femoral hernia repair and intra-operatively, appendix was found inside the hernia sac. The patient underwent open femoral hernia repair, and the appendix was pushed inside along with the sac contents (as the appendix was not found inflamed). The patient made good post-operative recovery and was discharged on the same day.

**Discussion:**

De-Garengeot hernia should be suspected in any patients coming with groin swelling, particularly if there's a possibility of incarcerated femoral hernia. CT scan can help to diagnose the condition pre operatively, but it is mainly found during operation. Management is surgical and depends on intra-operative findings.

**Conclusion:**

Despite being a rare case, a De-Garengeot hernia must be a differential diagnosis when evaluating a patient for a femoral hernia. Surgeons must be prepared for unexpected intraoperative findings.

## Highlights

• De Garengeot hernia is a rare condition where the appendix is found within a femoral hernia sac.
• It is usually diagnosed intraoperatively, as preoperative imaging like ultrasound may miss the diagnosis.
• This case involved an incidental finding of a non-inflamed appendix during elective femoral hernia repair.
• The appendix was reduced without appendectomy due to anatomical limitations and absence of inflammation.
• Surgeons should maintain a high index of suspicion for De Garengeot hernia, even in asymptomatic patients.

## 1 Introduction

Femoral hernias account for 4 % of all groin hernias and 30–50 % of patients may present with features of a strangulated hernia [1,2]. De-Garengeot's hernia was first described by French Surgeon Rene-Jacques Croissant De-Garengeot in 1731; however, the first appendicectomy in a De-Garengeot hernia was done by Hevin in 1785 [3]. Appendix has been found in 0.5–5 % cases of femoral hernia [1] and the incidence of appendicitis is even rarer, occurring 0.08 %–0.13 % of patients [2]. Due to its rare clinical presentation, preoperative diagnosis is challenging; it is mainly diagnosed intra-operatively [1,2,10]. Our case report describes an asymptomatic De Garengeot hernia diagnosed intra-operatively in an elective setting, with a conscious decision to preserve a non-inflamed appendix, guided by surgical feasibility and patient consent. Additionally, the case highlights the importance of careful pre-operative planning, the limitations of ultrasound, and the surgical judgment required when anatomical constraints prevent full visualisation of the appendix base. This case also states that the appendix can also be found incidentally during elective femoral hernia repair, reinforcing the need to consider this diagnosis even in asymptomatic patients. This case report has been reported in line with the SCARE checklist [19].

## 2 Case presentation

We present a case of a 79-year-old lady who was scheduled for elective day case surgery for a right femoral hernia repair. Her past medical history included cataract surgery, type 2 diabetes mellitus and vaginal prolapse. She had a right groin swelling for few years, which had remained asymptomatic apart from occasional mild discomfort while sitting or during physical activity.

During pre-operative assessment, she denied any nausea, vomiting and abdominal pain, fever or changes in bowel habit. Physical examination revealed an irreducible, soft, non-tender, non-erythematous lump which was lateral to her right pubic tubercle. There was no cough impulse. All other examination findings were normal. Her blood pressure, pulse were stable, and her preoperative laboratory tests, including white cell count (WBC 9.0 × 10^9^/L), haemoglobin (Hb 139 g/L) and renal function were within normal limits. She had a pre-operative ultrasound of right groin which showed a right sided femoral hernia. No further imaging was performed prior to surgery.

**Fig. 1:**
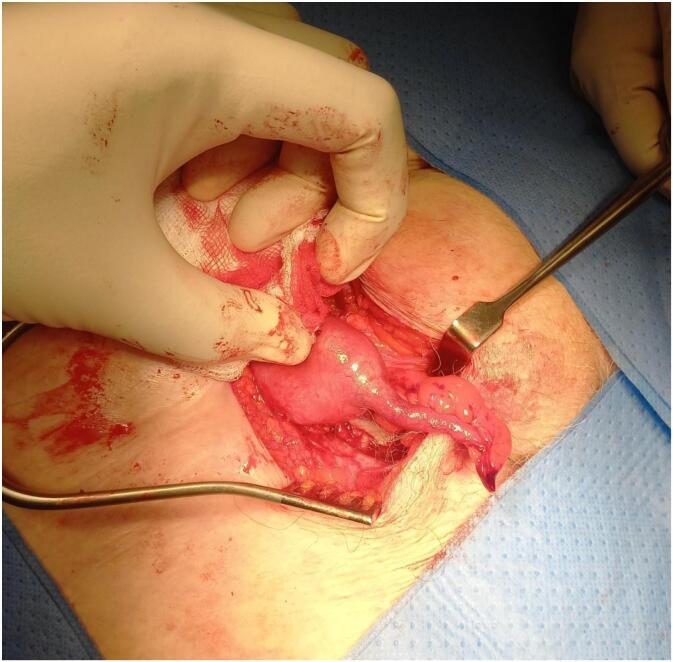
Appendix found inside femoral hernia.

During the day of operation, under general anaesthesia, procedure was started. Right groin incision was made. Hernia sac was identified and opened. Caecum and a non-inflamed appendix were found inside the sac (Fig. 1). Caecum contained hard stool and the appendix was macroscopically normal. Given the narrow femoral neck and limited operative field, performing an appendicectomy via the groin approach was deemed technically challenging and potentially unsafe due to risk of bleeding from the appendicular artery, which could have necessitated a laparotomy. Additionally, the patient had not been consented pre-operatively for an appendicectomy.

Following that, The Content of the hernia was pushed inside along with the appendix and the defect was closed with sutures. Local anaesthetic was given around the incision line. Post-operatively, she made an uneventful recovery. She was discharged on the same day after the operation.

## 3 Discussion

Femoral hernia sac mainly contains preperitoneal fat, bowel, omentum, etc. When an appendix is found inside a femoral hernia sac, it is called a De-Garengeot hernia [1]. Till now, fewer than 90 cases of De-Garengeot hernia have been reported [1]. The condition is more common in women as there is increased incidence of femoral hernia in women [2].

The presence of an appendix inside a femoral hernia can be explained by the appendix being pushed inside the femoral canal by large caecum or abnormal attachment of appendix to caecum secondary to embryogenic rotations [4,6]. Narrow neck of femoral hernia can cause appendicitis owing to extra-luminal compression [4]. It should not be confused with Amyand's hernia, where appendix is found within the inguinal hernia sac.

De-Garengeot hernia typically presents with an irreducible, tender groin lump along with abdominal pain [7]. Clinical examination could reveal abdominal pain, non-reducible hernia, localised signs of inflammation (erythema, warmth, tenderness) [8]. Patients seldom present with peritonitis as the inflamed appendix is separated from the peritoneal cavity by a narrow femoral neck [9]. Occasionally, as in our case, the patient may be asymptomatic; and the appendix inside the hernia can be found incidentally during intra-operative period. Absence of groin symptoms makes the diagnosis more challenging.

Establishing the diagnosis is difficult. The diagnosis is mainly made during operation, but few cases have been diagnosed pre-operatively through imaging [10]. Among the available imaging tests, CT scan is the gold standard to diagnose De-Garengeot hernia. Ultrasound scan can reveal bowel contents inside hernia sac [11]; but Abdominal CT scan has higher specificity and sensitivity [12]. Preoperative diagnosis and imaging are essential for planning the surgical approach in cases of De Garengeot hernia. Accurate imaging allows for better operative strategies and helps anticipate potential complications. In our case, the diagnosis was not made preoperatively, as the patient had a longstanding, asymptomatic groin swelling and underwent only an ultrasound.

Due to lack of reports in literature and its low incidence; there is no specific surgical guideline [4]. Surgical decision-making is therefore must be considered on the presence or absence of inflammation, patient comorbidities, intraoperative findings and surgeon's experience.

One common approach is open femoral hernia repair with appendicectomy, mainly for emergency cases [5]. The open techniques include the Lockwood (low), Lotheissen (transinguinal), and McEvedy (high) incisions; each offering different levels of access to the femoral canal and abdominal cavity [16]. One drawback of performing an open appendectomy via a groin incision is the potential need for a laparotomy if the base of the appendix cannot be safely visualized or accessed [16].

Alternatively, laparoscopic approaches, such as transabdominal pre-peritoneal (TAPP) or total extra-peritoneal (TEP) repair have been described [14,16]. Laparoscopic approach gives the advantage to explore abdomen allowing for both appendectomy and hernia repair in a minimally invasive manner [14]; but it should not be used in case of complicated hernias due to increased risk of complications and technical difficulty [14,15].

Primary suture repair is feasible for inflamed appendix; however, the use of mesh is often reasonable if the appendix is found normal [13]. Mesh is not preferred when the appendix is inflamed due to risk of infection [13].

The role of routine appendectomy in De Garengeot hernia remains debated. While some authors advocate for appendectomy regardless of the appendix's macroscopic appearance; considering the risk of future subclinical appendicitis and the potential need for re-operation [17]. Others caution against unnecessary appendectomy due to the increased risk of surgical site infection, particularly in elderly or nutritionally compromised patients [18]. Thus, intraoperative judgment remains critical, and management should be individualised based on operative findings and patient-specific factors. Reported post-op wound infection rate is 29 %, which is related to delay in diagnosis, old age and poor nutritional status [13,15].

## 4 Conclusion

De Garengeot hernia is a rare clinical entity, typically diagnosed intra-operatively, but as shown in our case, it can also present during elective femoral hernia repair. Surgeons should maintain a high index of suspicion, even in asymptomatic patients, especially elderly women. A key takeaway from our experience is the importance of intraoperative decision making; particularly when anatomical constraints make appendicectomy inappropriate. Currently, there are no standardised guidelines on the surgical approach, appendectomy indications, or mesh use, which highlights the need for further research and consensus-building. Until then, individualised management based on intraoperative findings and patient factors remains essential. Symptomatic patients should undergo prompt surgical management to avoid complications.

## Author contribution

Dr. Shoieb Hossain Mridha—Data collection, writing, study concept, literature review

Dr. Ayman El-shihaby—Writing, idea and literature review

Dr. Lakshmanan Arunachalam—Revising and Correction of case report, Supervision.

## Consent

Written informed consent was obtained from the patient for publication and any accompanying images. A copy of the written consent is available for review by the Editor-in-Chief of this journal on request.

## Ethical approval

Ethics approval is not required for case reports in Doncaster & Bassetlaw Teaching Hospitals NHS Trust as they are deemed not to be research.

## Guarantor

Dr. Lakhsmanan Arunachalam; Consultant, Colo-rectal Surgeon.

## Research registration number

N/A.

## Funding

Not applicable.

## Conflict of interest statement

All authors declare that there is no conflict of interest.
